# EmERGE mHealth Platform: Implementation and Technical Evaluation of a Digital Supported Pathway of Care for Medically Stable HIV

**DOI:** 10.3390/ijerph18063156

**Published:** 2021-03-18

**Authors:** Francisco J. Gárate, Paloma Chausa, Jennifer Whetham, Christopher Iain Jones, Felipe García, César Cáceres, Patricia Sánchez-González, Edward Wallitt, Enrique J. Gómez, on behalf of the EmERGE Consortium

**Affiliations:** 1 Biomedical Engineering and Telemedicine Centre, ETSI Telecomunicación, Center for Biomedical Technology, Universidad Politécnica de Madrid, 28040 Madrid, Spain; cesar.caceres@urjc.es (C.C.); p.sanchez@upm.es (P.S.-G.); enriquejavier.gomez@upm.es (E.J.G.); 2 Department of Sexual Health and HIV Medicine, Brighton and Sussex University Hospitals NHS Trust, BN2 5BE Brighton, UK; j.whetham@nhs.net; 3 Brighton and Sussex Medical School, BN1 9PH Falmer, UK; c.i.jones@bsms.ac.uk; 4 Infectious Diseases Department, Fundacio Privada Clinic per a la Recerca Biomedica—IDIBAPS, University of Barcelona, 08036 Barcelona, Spain; fgarcia@clinic.cat; 5 Computer Science Department, Universidad Rey Juan Carlos, 28933 Madrid, Spain; 6 Centro de Investigación Biomédica en Red, Biomateriales y Nanomedicina (CIBER-BBN), 28029 Madrid, Spain; 7 Podmedics, HA6 2UE Northwood, UK; edwallitt@me.com

**Keywords:** mHealth, patient empowerment, self-management, HIV, health technology assessment, co-design, model for assessment of telemedicine applications, feasibility, user acceptance, SUS, PREM

## Abstract

In this article, we described a new mobile-Health (mHealth) supported clinical pathway of care for people living with medically stable HIV in terms of platform acceptability, usability and technical feasibility. The EmERGE mHealth platform was codesigned with clinicians and the community, developed using Scrum agile methodology, integrated with hospital information systems and validated in a large prospective cohort study of 2251 participants. The evaluation of this new paradigm of care was conducted using a tailored Health Technology Assessment: the Model for Assessment of Telemedicine Applications. Usability and acceptability were assessed through the System Usability Score and a Patient Reported Experience Measure. The EmERGE platform was successfully deployed across diverse care settings in five European countries and used by 2251 patients and more than 20 clinicians for up to 30 months. Results from the formal evaluation demonstrated that the EmERGE platform is feasible and acceptable, with a high level of usability (median System Usability Score (SUS) 85.0%) and very positive patient-reported experiences (94.2% would recommend to a friend). The EmERGE platform is a secure and General Data Protection Regulation (GDPR)-compliant system with a complete set of functionalities that could be easily adapted to other clinical conditions, clinical sites and health systems thanks to its modular technical architecture.

## 1. Introduction

Electronic health (eHealth) solutions have demonstrated their potential to provide more personalised “citizen-centric” healthcare that is targeted, effective and efficient, facilitating access to care and improving patients’ quality of life [1]. eHealth refers to those healthcare services provided with the support of information and communication technologies (ICT). As part of eHealth, mobile health (mHealth) solutions, which imply the use of portable devices (smartphones, tablets and, even, wearables like smartwatches), make it possible to monitor and intervene remotely whenever and wherever acute and chronic medical conditions occur, as mHealth overcomes many of the traditional barriers of distance, time and cost, offering convenience and opportunities for care in nonclinical environments [2,3,4]. Based on this clinical evidence, organizations and associations globally have been developing recommendations and policy initiatives aimed at fostering and facilitating the uptake and wider development of eHealth and mHealth interventions [1,5,6,7]. As a result, the European mHealth market is valued at 5.40 billion euros in 2020 and is expected to reach 23.58 billion euros by 2025 at a Compound Annual Growth Rate of 34.29% during the forecast period 2020–2025 [8]. However, retention rates are still low for medical apps: 20% on day 1, 7% on day 7 and 3.5% on day 30, as are those related to health and fitness: 20.2% on day 1, 8.5% on day 7 and 4% on day 30 [9]. Clinical evidence, clinician’s proactivity, data privacy and the integration into provider healthcare systems are fundamental to more fully recognizing the value of mHealth apps and, therefore, to increasing user adoption [10,11,12], which may lead to improvement in patient self-management and retention in care [13]. Effective self-management is a central component of chronic disease control to optimize health outcomes. Evidence has been documented that successful self-management improves the self-reported health status and health-related quality of life while decreasing healthcare utilization and costs [14]. Retention in care is key in the maintenance of individuals’ health, especially in those clinical conditions that require long-term therapy, usually with multiple drugs, which have potential side effects and interactions that may influence patients’ adherence to treatment, like tuberculosis or HIV infection [15].

HIV now has a normal life expectancy in individuals who have access to testing, treatment and care, as in the European Union, where it is estimated that 2.5 million of people are living with HIV [16,17]. People living with medically stable HIV are currently seen by an HIV clinician every three–six months in accordance with the European guidelines [18]. The majority of individuals have an undetectable HIV viral load on antiretroviral treatment, and many are now seeking to reduce the impact of HIV on their lives—for example, those related to drug side effects, psychosocial issues, stigma or numerous hospital visits.

Recent interventions have demonstrated that mHealth can have a positive impact on clinical outcomes, as well as improvements in retention in the care for key populations [19] and newly diagnosed HIV-positive people aged less than 30 years [20,21], who have been historically difficult to reach with traditional interventions. The findings from systematic reviews on eHealth and mHealth interventions for HIV show that there has been an increase in the number of self-management mHealth interventions evaluated with people living with HIV (PLWH) and that these interventions have been shown to be effective, feasible and acceptable [22]. However, only a small proportion of the interventions identified were developed using a theoretical framework, and the majority of them offer a small number of limited functions, with not enough user involvement in the design and development phases, which may affect user acceptance. In addition, the reviews identified gaps around the linkage to care, retention in care and initiation of antiretroviral therapy [23]. In one paper [20], the authors reflected on the significant technical and operational challenges of implementing a mobile phone-based intervention in urban South Africa, emphasizing the need to investigate further for mHealth approaches to be effective. Similarly, investigators [22] have identified the need to develop, implement and evaluate more comprehensive mHealth interventions in order to address the self-management needs of PLWH. Specifically, the authors stated the need to focus on those who are stable during treatment, an under-researched and promising area in which previous experiences have demonstrated that web-based telemedicine approaches are feasible and safe tools for the management of stable HIV [24,25].

Within the EmERGE project [26,27,28], we have codesigned, implemented and evaluated a novel mHealth pathway of care for people living with medically stable HIV in five diverse clinic settings. The EmERGE mHealth platform integrates with hospital information systems to provide patients with secure access to their own data and to offer an alternative to face-to-face appointments as part of a person-centered package of care. It also allows clinics to manage their capacity, creating more time for clinicians to focus on those with more complex needs. We report here on the feasibility, usability and acceptability of introducing the EmERGE platform in a digital health pathway of care.

## 2. Materials and Methods

### 2.1. Technology Used

The platform consists of a web application that is installed in the clinical facilities and a mobile application, available on Apple and Google Stores. Apart from these two main components, two interfaces were developed for exchanging and managing data. The technologies involved in the development of the EmERGE platform were Ruby 2.3.3p222 (2016-11-21 revision 56859) (x86_64-linux) [29] and Ruby on Rails 4.2.10 [30] for both the web application and the two interfaces and Ionic 6.5.0 for the mobile application [31]. Ruby on Rails is an open-source, mature and well-maintained server-side web application framework written in Ruby. It is a Model–View–Controller framework, providing default structures for databases, web services and web pages. It encourages and facilitates the use of web standards such as JSON or XML for data transfer and HTML, CSS and JavaScript for display and user interfacing. The mobile application was built using a hybrid approach provided by the Ionic Framework, a complete open-source mobile UI toolkit for developing high-quality cross-platform apps for native iOS, Android and the web, using web technologies. Ionic is based in Apache Cordova, an open-source mobile development framework, which allows Ionic to build and deploy as a native app. The core of Cordova applications uses CSS3 and HTML5 for their presentation and JavaScript for the logic. HTML5 provides access to the underlying hardware of the mobile phone. For Android, Ionic supports Android 4.1 and up. For iOS, Ionic supports iOS 7 and up. Heroku is a cloud platform as a service supporting several programming languages. It lets the developer build, run and scale applications. Heroku [32] has been used to host several components of the EmERGE platform and the Heroku Command Line Interface 7.47.6 linux-x64 node-v12.16.2 to create and manage the Heroku apps directly from the terminal.

### 2.2. EmERGE Platform Development Methodology

The EmERGE platform was developed using Scrum, an agile methodology based on an iterative and incremental approach, delivering the software on a regular basis for feedback. Agile development is recommended for small-to-medium-sized projects [33], and many studies consider the agile method as a natural fit for the mobile app industry, although it is recommended to tailor the development process to better suit mobile apps’ peculiarities [34]. Numerous agile approaches for mobile software development have been proposed. In the literature, Extreme Programming and Scrum are the most common agile methods for mobile application development [33].

The development process followed produced incremental versions of the whole platform considering the feedback coming from the codesign process implemented by the EmERGE Consortium. The codesign process consisted of 3 phases of codesign workshops and semi-structured interviews [35,36]. In phase 1, 14 workshops and 22 interviews were conducted involving 97 PLWH and 63 clinicians. In this phase, participants were asked to reflect on the potentiality and risks associated with mHealth in the context of HIV treatment and care and on the functionalities that could support the self-management of HIV. Phases 2 and 3 aimed at capturing patients’ and clinicians’ experiences in the use of the platform to support continuous improvement. Thirteen workshops and 47 interviews were conducted during phases 2 and 3 involving 87 PLWH and 32 clinicians.

Different deployment environments were created to deal with the iterative development process: development, User Acceptance Testing (UAT) and production environments. A UAT allows final users to test the system and verify that the requirements and specifications are met. Finally, a ticketing system was used to better manage the support and maintenance activities during the formal evaluation and, therefore, guarantee the feasibility of the project.

### 2.3. EmERGE Platform Evaluation Methodology

A large prospective cohort study, undertaken in five European sites, was designed and performed to assess the impact of the mHealth supported pathway of care using a tailored Health Technology Assessment (HTA) process: the Model for Assessment of Telemedicine Applications (MAST) [37]. MAST summarizes and evaluates information about the medical, social, economic and ethical issues related to the use of telemedicine in a systematic, unbiased and robust manner.

The five clinical sites involved in the study were HIV clinics in Antwerp, Barcelona, Brighton, Lisbon and Zagreb. The general care of PLWH was very similar in the five clinical sites—specifically, in the follow-up of individuals living with stable HIV. PLWH who were participating in the study had visits at baseline and months 6, 12, 18, 24 and 30. At baseline, participants visited the hospital for a face-to-face visit and were introduced to the EmERGE platform. At six months, the individuals attended for bloods tests, as per the standard of care. The results were checked by a clinician and then pushed through to the patient’s mobile phone if the results were unremarkable. Visits at 18 and 30 months were conducted in a similar fashion. At 12 months and 24 months, the patients were seen by their clinical team in standard care.

As part of the formal evaluation, usability was assessed through the System Usability Score (SUS) [38], a scoring system that enables the evaluation of many interventions, including hardware, software, mobile devices, websites and applications. EmERGE participants were asked to complete the SUS after one year and two years of mHealth use. The SUS is a 10-item Likert scale (1–5, strongly disagree to strongly agree) assessment of usability that includes the following questions:

1. I think that I would like to use this system frequently.
2. I found the system unnecessarily complex.
3. I thought the system was easy to use.
4. I think that I would need the support of a technical person to be able to use this system.
5. I found the various functions in this system were well-integrated.
6. I thought there was too much inconsistency in this system.
7. I would imagine that most people would learn to use this system very quickly.
8. I found the system very cumbersome to use.
9. I felt very confident using the system.
10. I needed to learn a lot of things before I could get going with this system.

For items 1, 3, 5, 7 and 9, the contribution is the scale position minus 1. For items 2, 4, 6, 8 and 10, the contribution is 5 minus the scale position. The score is calculated as 2.5 × (sum of item contributions). Therefore, the resulting score ranges from 0 to 100, with a higher score indicating greater usability [39]. A score over 68 is considered above average [40].

Acceptability was assessed by means of a Patient-Reported Experience Measure (PREM) completed by participants at month 12 and month 24. This paper reports the results of two measures included in the PREM, as they were the questions related to the use of the EmERGE platform as part of the mHealth supported pathway:

- “How would you rate your overall satisfaction with the EmERGE service?” (Excellent/Good/Satisfactory/Poor/Very poor).
- “Would you recommend the EmERGE service to a friend?” (Yes/No).

In addition to the acceptability and usability assessment, this article describes the usage data to analyze the use, performance and operation of the EmERGE platform both from the PLWH and the clinical and administrative staff perspectives. It also allows comparisons of the use of the platform in different clinical sites.

## 3. Results

### 3.1. EmERGE Platform Specification

The objective of the EmERGE platform is to provide the relevant, timely and secure delivery of healthcare data to people living with stable HIV infection through the use of novel mHealth tools. These healthcare data include medication data (name and information about the drug, dose, frequency and date of commencement); appointments data (date, time and type of the appointment) and blood test results (current and historical data and evolution graphs). The data are sent along with a message from the clinician that provides the patient with the necessary information to interpret and understand the data. The main users of the EmERGE platform are PLWH and the clinical and administrative staffs of the clinical sites involved. The use cases and the specific requirements of the platform were determined through a codesigned work with PLWH and clinicians [35,36]. Table 1 and Table 2 summarize the functional and nonfunctional requirements, respectively, as codesigned with clinicians and the community.

### 3.2. Architecture

**Figure 1:**
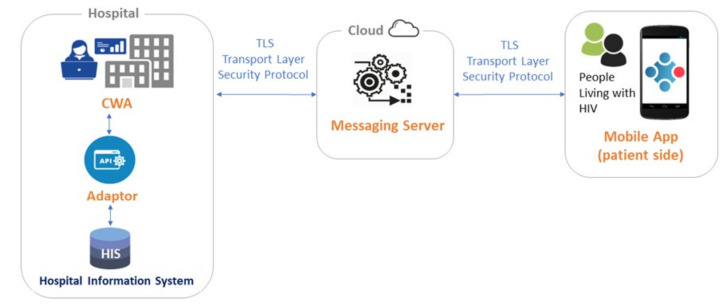
EmERGE platform architecture. CWA: Clinical Web Application.

The EmERGE platform consists of four main components: the Adaptor, the Clinical Web Application (CWA), the Messaging Service (MS) and the Mobile Application. This group of software allows clinical data to be gathered from the hospital network, formatted appropriately, sent to and displayed on patient devices. The basic structure of the EmERGE platform and the interaction of the four key elements is shown in Figure 1.

The Adaptor provides a single Application Program Interface (API) for the CWA to extract the data via an interface with each hospital database. The Adaptor ensures that the data are in a single format for onward processing, facilitating the integration with different clinical systems. In the context of this study, each clinical site had a very different way of obtaining data: Brighton and Lisbon both used API-based systems, whereas Zagreb and Antwerp both provided SQL Server databases from which to gather data. The Adaptor integrates these with different demographic and clinical information systems by adjusting the correct set of models for gathering data. The implementation of the Adaptor was made in close collaboration with the IT staff of each hospital. The CWA is hosted in the hospital network and provides clinical users with a web application to register and manage patient users and their mobile devices and to provide executive control over the delivery of relevant information to mobile devices. The MS is a cloud-based API sitting between the CWA and the Mobile Application that stores encrypted data around medication, blood test results and appointments for specific individuals. The MS is responsible for delivering these data to the Mobile Application on demand, so that this information is not stored on the patient mobile device. The Mobile Application receives user specific information from the CWA via the MS and provides app users with access to their own data.

### 3.3. User Interfaces and Graphical Design

**Figure 2:**
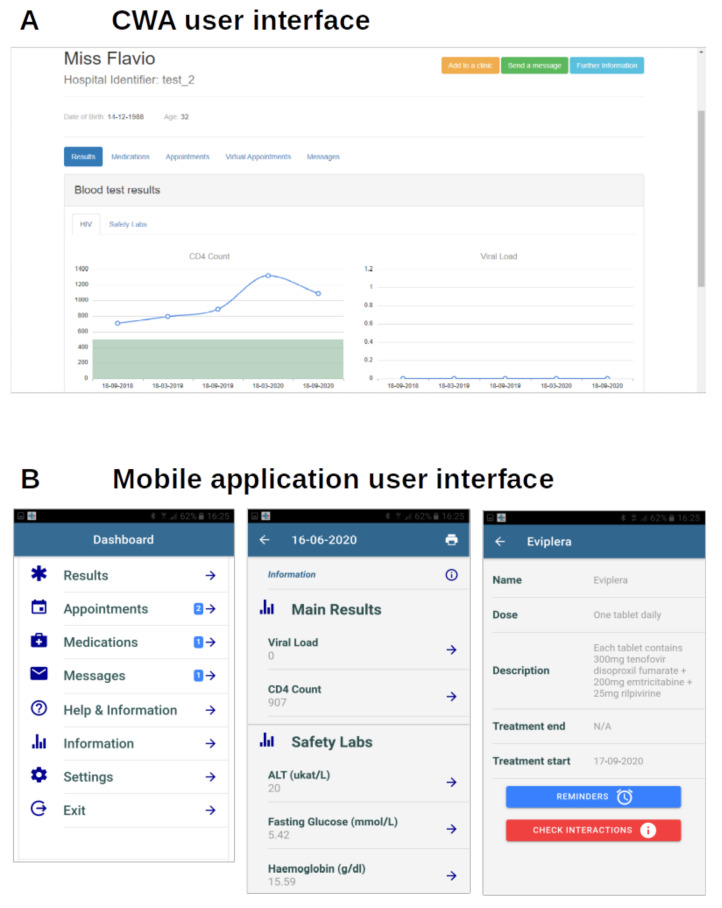
CWA (**A**) and Mobile Application (**B**) User Interfaces.

The EmERGE platform offers two main User Interfaces: the CWA for clinicians and the Mobile Application for PLWH. The CWA was built using Bootstrap-Sass 3 for the graphical design. Bootstrap is a popular open-source framework that offers web designers many functions that facilitate development and accelerate site building. This version of Bootstrap uses Sass (Syntactically Awesome Style Sheets) as the preprocessor scripting language that is interpreted or compiled into CSS. The Mobile Application also uses Sass for the graphical design. Figure 2 shows the CWA and the Mobile Application interfaces.

### 3.4. Security and Privacy

Security is a key factor for our patient group and for their clinicians. The CWA within each site is regarded as a “walled-off data silo”. It exists within the existing IT infrastructure of the clinical site and should not be open for external access. As such, unless connected via a Virtual Private Network, it is not possible to access to the CWA from outside of the hospital network. All clinical and demographic data stored locally within the CWA’s local database are encrypted to a minimum of 256-bit Advanced Encryption Standard (AES). The transmission of data outside of the hospital network, from the CWA to the Mobile Application, is encrypted over Transport Layer Security (TLS) and includes no patient identifiable information. As an additional security measure, the clinical information is not stored on the patient’s mobile device at any point.

Strong authentication procedures were designed in order to guarantee confidentiality and privacy. In this regard, a one-time password was generated by the clinical site personnel during the patient registration procedure and, once communicated to the patient, was permanently deleted. This password was required during the installation and setup of the Mobile Application, as well as an additional passcode defined by the patient, and used to sign into the Mobile Application from that moment onwards.

### 3.5. Evaluation of the EmERGE platform

#### 3.5.1. Usage Data

The EmERGE platform was successfully deployed in five clinical sites in Europe during 2017: the sites were integrated sequentially between April 2017 and November 2017. The patients (2251) used the platform by downloading and installing the Mobile Application: 309 patients in Zagreb, 565 in Brighton, 549 in Barcelona, 249 in Antwerp and 579 in Lisbon. Enrollment closed in October 2018, with the follow-up continuing for a minimum of 12 months. Of the individuals enrolled in the study, 2048 (91.0%) were male; the median age was 43.0 years (interquartile range (IQR) 37.0 to 51.0 years). Eighty-two (3.6%) withdrew from the study within the 12 months of follow-up, with a further 84 (3.8%) by 30 months (a total of 166 withdrawals). There were no security incidents and only three major technical incidents and bugs identified during the course of the cohort study (April 2017–October 2019): (1) blood test result evolution graphs not being displayed, (2) Antwerp server not available for one week and (3) automatic log out of the Mobile Application. These technical issues were solved with no significant impact on the study, although the automatic log out issue caused the withdrawal of several patients.

**Figure 3:**
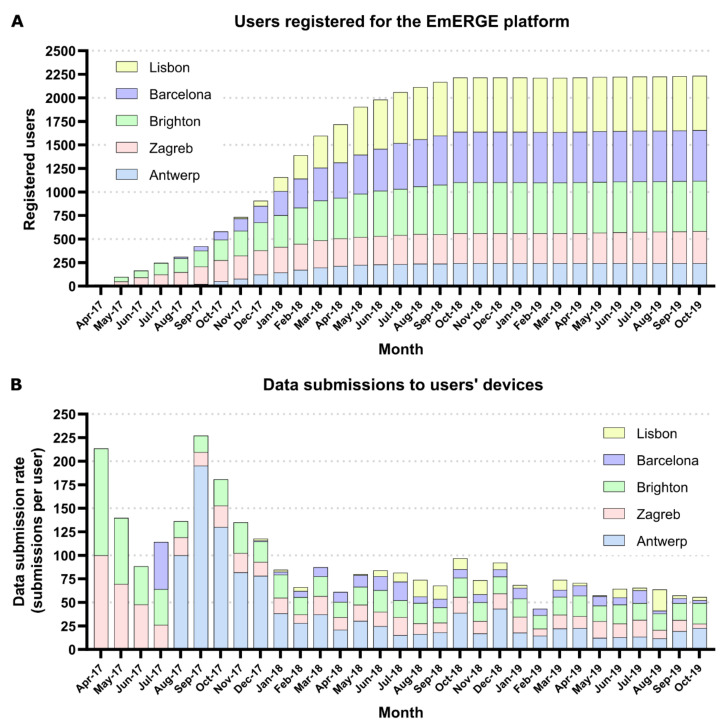
(**A**) Evolution of the number of patients registered for the EmERGE, and (**B**) data submissions to users’ devices at each clinical site during the course of the cohort study.

The usage of the EmERGE CWA in each clinical site is described in Figure 3 by the number of patients registered on the EmERGE platform (A) and the number of data submissions to the users’ Mobile Application (B) at each clinical site during the course of the cohort study. The number of data submissions was weighted according to the total number of patients registered on the platform in each site to allow comparisons among the different clinical sites.

**Figure 4:**
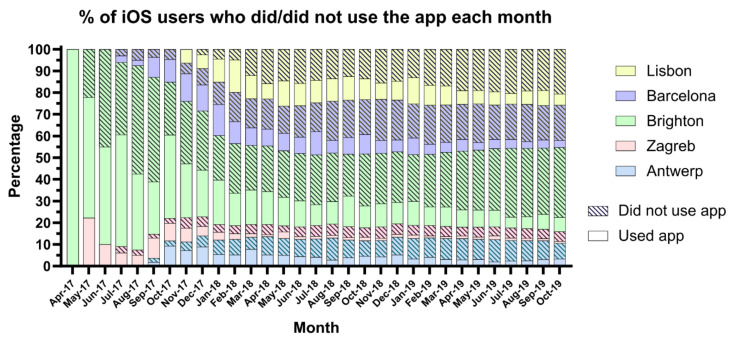
% of iOS users who did/did not use the app each month at each clinical site.

For the EmERGE Mobile Application, this paper reports the usage data of the iOS users, as the historical usage data from the Android users are not available. This is due to a modification in the statistics reports carried out by Google in July 2019 that impeded the retrieval of consistent data for the analysis. From the App Analytics tool available in Apple Store Connect, we can state that at least 33.8% of EmERGE participants used iOS: 54.8% in Brighton, 33.6% in Lisbon, 32.9% in Antwerp, 27.3% in Barcelona and 11.8% in Zagreb. There were a total of 840 installations and 86 uninstallations during the course of the cohort study. Eighty-seven point one percent of participants maintained the app installed in their devices, ranging from 73.4% in Antwerp to 96.1% in Brighton. Figure 4 shows the monthly distribution of the number of iOS devices with at least one session during the course of the study, i.e., the percentage of iOS users who did and did not use the app each month at each clinical site. It is important to note that the App Analytic tool only reported data from those users that consented to data sharing.

#### 3.5.2. Impact on Acceptability and Usability

All PLWH enrolled in the study were asked to complete the SUS questionnaire and a questionnaire on their experience with the new mHealth-supported pathway at month 12 and month 24. The use of different platforms (Android vs iOS) did not influence the usability results and the experiences reported, as the EmERGE Mobile Application provided identical functionalities and graphical user interfaces thanks to the cross-platform approach followed.

**Figure 5:**
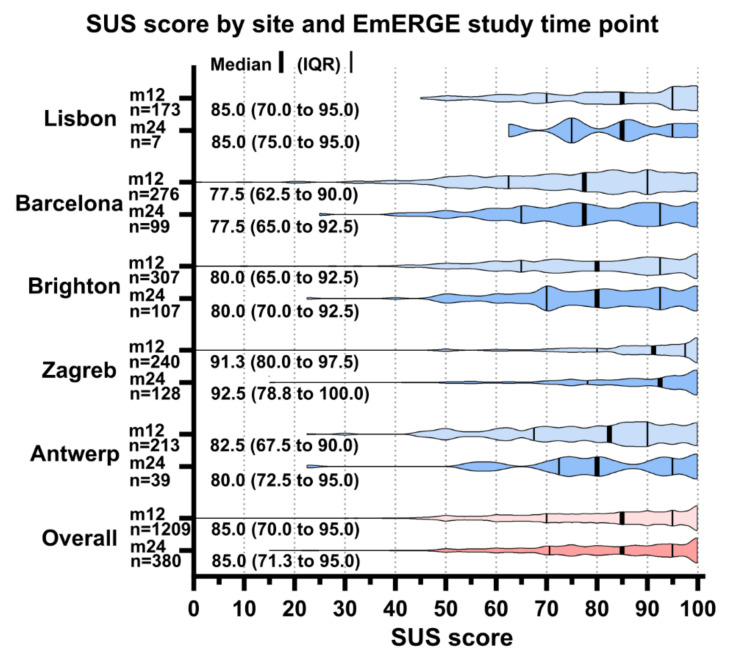
System Usability Score (SUS) score by site and EmERGE study time point. IQR: interquartile range.

The SUS was considered complete if a SUS score could be calculated (all items were entered). The SUS was completed by 1209 participants at month 12 and by 380 at month 24. Limited data was available for Lisbon for month 24 (seven participants). Figure 5 shows the SUS score by site and EmERGE study time point.

The SUS scores were high at all sites at both month 12 and month 24, with the medians ranging from 77.5 in Barcelona at month 12 and month 24 to 100.0 in Zagreb at month 24. Within the sites, the scores between the time points were very similar. Overall, the median (IQR) SUS score at month 12 was 85.0 (70.0 to 95.5) and 85.0 (71.3 to 95.0) at month 24.

**Figure 6:**
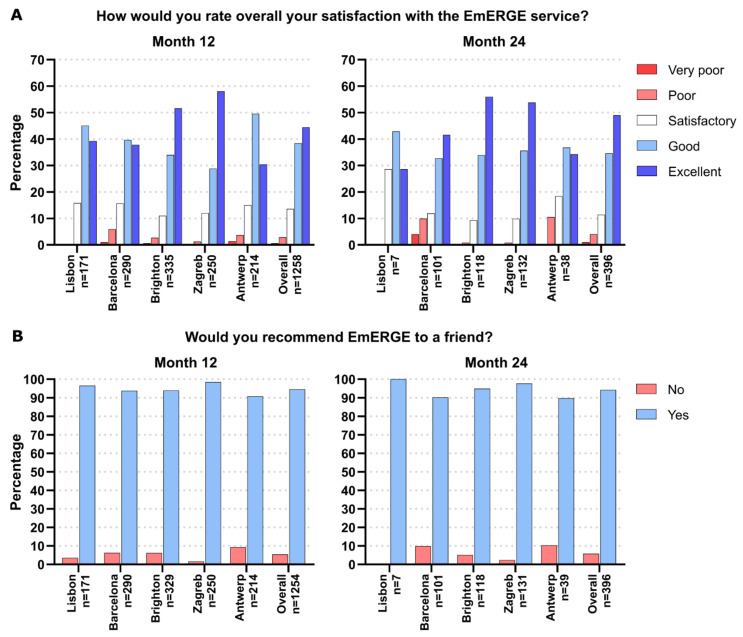
Distribution of the responses related to satisfaction with the EmERGE service at month 12 and month 24 across the sites.

The PREM was collected at month 12 and month 24. Here, we report the results of two measures included in the PREM questionnaire, as they are the questions related to the use of the EmERGE platform as a part of the mHealth-supported pathway. Figure 6 shows that participant satisfaction with the EmERGE service was high at all sites at both time points, with 82.8% and 83.6% of participants rating it either “Good” or “Excellent” overall at month 12 and month 24, respectively. Ninety-four point six percent of the participants would recommend EmERGE to a friend at month 12 and 94.2% at month 24. Overall, this ranged from 89.7% in Antwerp at month 24 to 98.4% in Zagreb at month 12 (excluding the 100% at Lisbon at month 24, as this was based on only seven participants).

## 4. Discussion

This article provides clinical evidence on the implementation of mHealth interventions, demonstrating that this digital supported pathway of care is considered feasible and acceptable by people living with stable HIV and their healthcare providers. In this regard, a large and high-quality cohort study has been designed and implemented and a structured and comprehensive HTA framework has been used, MAST, which provides a scientific robust approach based on rigorous data and enables evidence-informed decision-making. MAST fosters the involvement of the users in the assessment process enhancing the acceptability and usability of the mHealth applications developed. In the EmERGE project, a strong emphasis has been placed on the codesign phase, which has resulted in a person-centered digital solution. The benefits of people-centered approaches are well-documented, and policymakers have recognised the need to promote them in order to provide health services that are responsive to individuals and communities [1,41,42]. Another important characteristic of the EmERGE platform is the capability of integrating with hospital information systems to provide PLWH with secure access to their own data. All of these characteristics may have contributed to the high perceived value of the platform by end users, as stated by the acceptability and usability assessment results. The SUS scores at all sites at months 12 and 24 showed a high level of usability, with medians ranging from 77.5 in Barcelona at month 12 and month 24 to 100.0 in Zagreb at month 24, where 77.5 could be described as “Excellent” and 100 as the “Best Imaginable” [40]. The satisfaction with the EmERGE service was high at all sites at both month 12 and month 24, according to the PREM results. The differences between clinical sites’ results could be motivated by their previous experience with digital pathways, as well as the benefits perceived by the end users, such as, for example, Barcelona, where there was already an eHealth system with a very complete set of functionalities [24,25], or Zagreb, with no digital background, where the introduction of the EmERGE platform has improved the reporting of blood test results with a more systematic procedure.

The usage data showed that the EmERGE platform has been used uniformly in each clinical site once the patient population was stable (from June/July 2018), although Barcelona and especially Lisbon professional users carried out fewer data submissions, considering the participants recruited in those sites. With regards to the use of the Mobile Application, the participants from Brighton represented the highest number of iOS users and Zagreb the fewest. The participants from Antwerp, Barcelona and Lisbon had similar distributions. All of the iOS users showed similar usages of the Mobile Application from November 2017. The lack of usage data availability for Android users represents a limitation to this study and should be taken into account when interpreting the results. Additional learnings could be extracted from the usage data if further data analytics tools would have been available, such as, for example, data usage patterns and the functionalities more frequently used. However, privacy issues should be considered before including these kinds of tools, as they may influence user acceptance. Despite these limitations, and taking into account the information coming from the usage data and the withdrawal rate, it could be inferred that the app retention was high, as well as the retention in care during the cohort study.

The clinical outcomes from the formal evaluation [26] and the smooth running of the EmERGE platform during the course of the cohort study demonstrated the clinical and technical feasibility of the EmERGE pathway overcoming all technical and operational issues reported by previous experiences [20,21]. The availability of the Mobile Application across common platforms and the use of access points to download the app (Google and Apple Play Stores) facilitated the recruitment of the study participants, as well as the quality of the app, thanks to the verification procedures provided by these platforms. The use of agile methodologies and cross-platform technologies allowed a quick adaptation of the platform to the evolving needs coming from the codesign process and the study implementation. The availability of different deployment environments and the use of a ticketing system were demonstrated to be useful to deal with both the iterative development process and the support and maintenance activities during the course of the study. Moreover, there were no security breaches and few technical incidents, despite the large group of users (2251 participants and more than 20 clinicians and administrative staff) and the long evaluation period (30 months).

The EmERGE platform is in compliance with the EU Digital Single Market strategy, which promotes citizens’ secure access to health data, the full-scale deployment of new care models, the use of HTA and the utilization of interoperable digital solutions for the delivery of efficient and cost-effective care. In this respect, the EmERGE platform is a General Data Protection Regulation (GDPR)-compliant tool [43] that ensures secure access to health data by the exchange of pseudo-anonymous and unreadable data and provides strong and reliable authentication procedures. Additionally, the modular technical infrastructure proposed allows the future uptake of the EmERGE platform by new clinical sites and new health systems by adjusting the Application Programming Interface adapter to the specific new site IT system characteristics. The mHealth platform could also be easily scaled up and transferred to different clinical domains.

## 5. Conclusions

mHealth technology is being used increasingly and may be a leading tool in future healthcare paradigms in which personalized health and care, and the use of digital tools for citizen empowerment and for person-centered care, in compliance with data protection rules, acquire special relevance [7]. There is a large body of evidence through Randomized Clinical Trials and systematic reviews that mHealth is an effective mechanism for innovative healthcare strategies and a promising solution to provide efficient, cost-effective and patient-centered care, both in terms of prevention and treatment [44]. In the field of HIV, the successful scale-up of antiretroviral therapy globally gives mHealth applications the potential to transform HIV care beyond viral suppression in terms of comorbidity management, health-related quality of life and patient-reported outcomes [45]. However, there are arguments that further rigorous studies are needed to assess the outcomes and guide the implementation of personalized mHealth interventions aimed at enhancing the uptake and acceptability in routine practice that is fundamental for enabling a continuity of care [7]. In this regard, and thanks to the funding received from the European Union’s Horizon 2020 research and innovation programme, this article provides clinical evidence on the implementation of mHealth interventions through the assessment of a novel mHealth pathway of care for people living with medically stable HIV. The EmERGE platform supporting the digital pathway provides person-centered services, complies with data protection regulations and has been shown to be feasible and acceptable by end users. A not-for-profit company has been created with the aim of expanding the EmERGE paradigm of care to other clinical sites in Europe and supporting new research experiences to gain clinical evidence. New clinical sites have already shown their interest in implementing the EmERGE clinical pathway, and the research intervention is being defined in the context of the pre-exposure prophylaxis, a successful intervention to prevent HIV infection. Future works will include, among others, the development of a video conference and a messaging system to provide synchronous and asynchronous communication between patients and healthcare providers, a module for medication collection, the migration of the technology to React Native and the implementation of recommended standards for exchanging Electronic Health Records across the EU (HL7 standard), which will enhance the interoperability capabilities of the EmERGE platform, facilitating digital scale-ups and fostering the large-scale deployment and uptake of the EmERGE platform.

## Figures and Tables

**Table 1:** Summary of the main EmERGE platform functional requirements. PLWH: people living with HIV.

Functionality	Description	User
Authentication	Users are required to login into the platform on each use	All users
Patient/device registration	Registration of patients and the associated mobile devices	Administrators and clinicians
All patient visualization	Search and filtering of all registered patients	Administrators and clinicians
Patient data visualization	Overview of each patient user: current medication list (name, dose/frequency and date of commencement); blood test results (graphically and in a table format) and appointment lists	Administrators and clinicians
“Virtual clinics” appointment management	Send data to the mobile application; rebook the patient for a future revision; pause the patient in the system	Administrators and clinicians
Messaging	Messaging from Hospital clinicians	All users
FAQ management	Provision of Frequently Ask Questions	Administrators
User management	Creation and elimination of users and assignment of roles	Administrators
Medication visualization	Visualization of medication list (name of medication, dose/frequency and date of commencement). Link to external Liverpool HIV drugs interaction webpage	PLWH
Blood test results visualization	Visualization of blood test results: current and historical data; evolution graphs	PLWH
Appointments	Visualization of next appointments. Integration with patient mobile calendar	PLWH
Help desk and information	Support and access to FAQs	PLWH
Application Settings	Erase application data, change passcode, change security question, ask for a new password, study withdrawal	PLWH

**Table 2:** Summary of the main EmERGE platform nonfunctional requirements.

Requirement	Description
Usability	Easy to be used and learnt, easy to be remembered, efficient and able to generate satisfaction in the users. Intuitive interfaces, readable and adapted to users, different web browsers and device resolution screens
Adaptability	Platform to be introduced in systems of different hospitals. It must be easy to implement and easy to readapt to different characteristics and requirements
Scalability	System architecture should be designed to appropriately handle increasing and decreasing workloads and prepared for a rapid expansion in number of clinical sites and number of users
Security	Information should be sent in a secure way. Stored data only accessible by authorized health staff. System only accessible to registered and validated users
Performance	The response time in operations must be the expected time of any web application
Multiplatform	The platform should be accessible through mobile applications (iOS and Android) or any of the existing web browsers depending on the role of the user

## Data Availability

The data are not publicly available due to the conditions of individual patient consent.
